# How healthy is it to consume soft and energy drinks? – primary school pupils’ opinions from Hungary

**DOI:** 10.1093/eurpub/ckac131.234

**Published:** 2022-10-25

**Authors:** J Girán, K Girán, D Ormándlaky, Z Kollányi, I Kiss

**Affiliations:** 1 Department of Public Health, University of Pécs, Medical School, Pécs, Hungary; 2 Bachelor Programme, Eötvös Loránd University, Faculty of Education and Psychology, Budapest, Hungary; 3 Directorate, Kodály Z. Catholic Primary School and Kindergarten, Komló, Hungary; 4 Department of Economics, Eötvös Loránd University, Faculty of Social Sciences, Budapest, Hungary

## Abstract

**Background:**

The consumption of soft drinks and energy drinks (SaED) in EU27 has hardly changed over the past decade. In contrast, it grows massively in Hungary, mainly among youths. In 2021, our research group studied the causes of the growing SaED consumption of primary school pupils. Now, we deliver our key findings on how pupils consider the health impacts of SaED consumption.

**Methods:**

The study took place in a primary school in Komló, Hungary, in a mixed methods design: 157 pupils aged 10 to 15 filled out a survey, which we analyzed using descriptive statistical methods and independency tests. Besides, focus groups were conducted involving three stakeholder groups: a) school pupils; b) pupils’ parents; c) school teachers (39 respondents in total).

**Results:**

Every fifth pupil consumes soft drinks daily and every third more than once a week. 28.1% of pupils consume energy drinks with some regularity, the majority more than once a week. Of those who think that soft drinks are rather unhealthy, 37.2% consume them daily or several times a week. Of those who believe that energy drinks are rather unhealthy, 24.6% consume them with some regularity. The focus groups confirmed that pupils perceive SaED consumption as accepted and “normal” behavior, and for the majority, the health consequences of the consumption are not a point.

**Conclusions:**

Although many pupils are aware of the health risk of SaED, they still drink them regularly. The general social acceptance of SaED consumption and the influential marketing of these products both support this attitude. To affect consumers’ behavior, messages on how “unhealthy” SaED are does not seem adequate: a change in community acceptance is needed.

**Key messages:**

• The knowledge of adverse health effects of soft and energy drinks itself won’t reduce consumption as long as its social acceptance is high.

• Preventive interventions should apply similar tools that have made soft and energy drink consumption popular and accepted to reverse recent trends.

